# Determinants of COVID-19-related hospital and ICU admissions in the region Haaglanden, The Netherlands: a cross-sectional study

**DOI:** 10.1186/s12889-025-23364-1

**Published:** 2025-07-02

**Authors:** K. B. Bingöl, I. Meulman, K. R. M. Wassing, I. M. van der Meer

**Affiliations:** 1 Department of Epidemiology and Policy advice, Public Health Service of Haaglanden, The Hague, The Netherlands; 2 Center for Health, Care and Society, National Institute for Public Health and the Environment, Bilthoven, The Netherlands; 3 Tilburg School of Social and Behavioral Sciences, Tranzo, Tilburg University, Tilburg, The Netherlands; 4 Department of Infectious Diseases, Public Health Service of Haaglanden, The Hague, The Netherlands

**Keywords:** COVID-19, Hospital and ICU admission, Neighborhood disparities, Determinants of health

## Abstract

**Introduction:**

The COVID-19 pandemic has been a global health crisis since late 2019. By the end of 2021, the Netherlands reported over 3 million cases, leading to significant hospital and Intensive Care Unit (ICU) admissions. This study investigated the impact of demographic, socio-economic, health, including vaccination coverage, and neighborhood characteristics on COVID-19-related hospital and ICU admissions, with a focus on neighborhood differences in the region Haaglanden, the Netherlands.

**Methods:**

This cross-sectional study included residents aged 25–79 years from the region Haaglanden. Data were stratified across three COVID-19 waves. Individual-level registry data from 2020 to 2021 were used, covering demographics, socio-economic factors, health information and neighborhood characteristics, linked to COVID-19-related hospital and ICU admissions. Multivariable logistic models were conducted per wave to estimate the odds of both COVID-19-related hospital and ICU admissions.

**Results:**

More than 700.000 inhabitants from the region Haaglanden were included per wave. COVID-19-related hospital admissions were 0.08% (*n* = 571) in the first wave, 0.40% (*n* = 2.865) in the second, and 0.17% (*n* = 858) in the third wave. ICU admissions were 0.02% (*n* = 159) in the first wave, 0.07% (*n* = 530) in the second, and 0.03% (*n* = 192) in the third wave. Hospital and ICU admission odds were higher among older individuals, males, lower-educated individuals, those of Moroccan origin, residents with lower income and wealth, poor physical health and those living in low socio-economic neighborhoods. In the third wave, neighborhoods with vaccination coverage below 60% had the highest rates of hospital and ICU admissions.

**Conclusion:**

This study highlighted that individual and neighborhood factors were associated with a higher risk of COVID-19-related hospital and ICU admissions, with the individual risk factors often concentrated in neighborhoods with low socio-economic status scores. Public health strategies should focus on high-risk individuals and incorporate tailored interventions, while early identification of disadvantaged areas is key for effective resource allocation and reducing disparities during future outbreaks.

**Supplementary Information:**

The online version contains supplementary material available at 10.1186/s12889-025-23364-1.

## Introduction

The coronavirus disease 2019 (COVID-19) pandemic, caused by SARS-CoV-2, created an uncommon global health challenge since its emergence in late 2019 [1, 2]. By December 31, 2021, the Netherlands had reported over 3 million cases in total out of a population of 17,590,672, resulting in approximately 89,000 hospital admissions and 16,000 intensive care unit (ICU) admissions [3, 4].

As the pandemic spread worldwide, it became evident that the severity of COVID-19 varied significantly among populations and regions, with some areas experiencing a disproportionately higher burden of hospital and ICU admissions. Studies indicated that there are risk factors for these admissions, which are influenced by individual and neighborhood characteristics, such as health, socio-economic, environmental, and racial differences [5, 6]. Additionally, research in European countries with universal healthcare showed that non-native groups and individuals with lower socio-economic status (SES) are at higher risk of COVID-19-related hospital admissions, ICU admissions, and mortality compared to native populations and individuals with higher SES [7].

Similar results were reported in the Netherlands [8–11], showing that the proportion of COVID-19-related hospital admissions was higher among men, ethnic minority groups, and older age groups. Research in the Netherlands also showed that COVID-19 vaccines were highly effective in preventing hospital and ICU admissions, with most hospitalizations occurring among unvaccinated individuals in the Netherlands [12]. In addition, individuals in the lowest income group had a lower chance of being tested and had a higher risk of testing positive [3]. This group also faced significantly higher rates of hospital and ICU admissions, which resulted in a nearly fourfold increased risk of mortality from COVID-19. Furthermore, individuals with a country of origin other than Dutch were more likely to have co-morbidities, which increased their risk of severe COVID-19 infection [14–16]. Moreover, differences in living conditions, such as lower wealth, larger household composition, lower household incomes, and living in densely populated urban areas, also increase the risk of hospital admission [11, 17]. Evidence also showed that living in low-SES neighborhoods nearly doubles the likelihood of COVID-19-related hospital admission, compared to individuals living in high-SES neighborhoods [10]. Given the impact of residential location on health, it is relevant to investigate transmissible infectious diseases at the neighborhood level, as infections frequently spread within close social networks and the surrounding environment. However, the complex relationships between social, demographic, and health characteristics at individual and neighborhood-levels in relation to COVID-19 outcomes, especially hospital and ICU admissions, remain understudied [18, 19].

Due to its unique characteristics and composition, the region Haaglanden presents a particularly interesting population for investigating the individual and neighborhood-level characteristics of COVID-19-related hospital and ICU admissions. The region Haaglanden, located in the west of the Netherlands, comprises nine municipalities with a total population of 1,1 million. The region is a densely populated, multicultural area with significant socio-economic diversity, including major urban center such as The Hague [20]. The region’s demographic and socio-economic diversity contributes to disparities in disease burden, disproportionately affecting individuals with migration backgrounds, various age groups, and different socio-economic statuses. Moreover, there are notable health differences between municipalities and their associated neighborhoods [20]. These vulnerabilities are further exacerbated by demographic shifts, including an aging population and increasing socio-economic health disparities. Particularly in the context of potential infectious disease outbreaks such as COVID-19, this poses a significant challenge to the continuity of healthcare services in the region.

In the Netherlands, the COVID-19 response was centrally coordinated by the national government and the National Institute for Public Health and Environment (RIVM) [21]. All regions, including region Haaglanden, followed national guidelines, such as lockdowns, social distancing measures, testing and vaccination strategies. Furthermore, the pandemic was marked by waves, each characterized by substantial contextual differences. While local implementation may have varied slightly, the core strategies remained consistent.

To date, research on the combined influence of demographic, socio-economic, health and neighborhood characteristics is scarce. This study aims to investigate the association between these various characteristics, including vaccination coverage, and COVID-19-related hospital and ICU admissions, with a specific focus on neighborhood differences in the region Haaglanden, the Netherlands. The added value of the current study lies in the diverse composition of neighborhoods within Haaglanden and the integration of various characteristics, which may help (regional) policymakers and experts in infectious diseases to improve pandemic preparedness plans, for instance through better control of infection outbreaks in the future.

## Research methods

### Study population and design

This cross-sectional study included residents aged 25–79 years old from the region Haaglanden. Inhabitants living in institutionalized household and those aged 80 years and older were excluded, as the health-seeking behavior of elderly and institutionalized individuals may be influenced by different mechanisms and care arrangements [22]. In particular, older adults residing in long-term care facilities were less frequently admitted to hospitals, as care was often provided within the facility itself. Furthermore, some individuals in this age group opted not to be hospitalized due to personal preferences or advance care directives. Including this group could therefore have introduced bias, as their hospital admission rates and associated determinants would likely differ from those of community-dwelling adults due to heterogeneity within this group. All individuals under the age of 25, primarily youth and students, were also excluded due to a weaker correlation between income, education, financial wealth, and their socio-economic position [23]. All analyses were stratified by the three different COVID-19 waves in the region Haaglanden, based on the number of COVID-19 infections and hospital admissions in the region Haaglanden. The first wave extended from February 24, 2020, until July 12, 2020. The second wave extended from July 13, 2020, until July 4, 2021, and the third wave extended from July 5, 2021, until January 2, 2022. To account for the substantial contextual differences between the three COVID-19 waves, including virus variants, public health measures, vaccination availability, and population behavior, separate models were used for each period. For each wave, the most recent population count as of the wave’s start date was used to ensure that the analyses reflected the actual population at risk during that period. Because populations change over time (in example through aging, migration, or mortality), the eligible population was updated at the start of each wave to account for such demographic changes.

### Data sources

Routinely collected pseudonymized individual-level registry data from the years 2020 and 2021 were used. Individual characteristics, including age, sex, educational level, country of origin, disposable household income, financial wealth, and household size, were derived from Statistics Netherlands’ registry data. These individual characteristics were linked at the individual-level to COVID-19-related hospital and ICU admissions from Dutch Hospital Data (DHD). This linkage was performed by Statistics Netherlands, using a pseudonymized personal identification number that is consistently available across registries. This identifier enables a deterministic linkage of different databases at the individual level. The pseudonymization is based on social security number when available. If this number is not available, a combination of variables is used, including sex, date of birth, date of death, postal code, and address. For neighborhood-level characteristics, physical health, the socio-economic status score per neighborhood, and vaccination coverage were used. Including indicators at both the individual and neighborhood levels allowed for the assessment of the additional association of neighborhood-level socio-economic characteristics alongside individual characteristics. Physical health was derived from the 2020 Health Monitors for Adults and the Elderly, conducted by Municipal Public Health Services, Statistics Netherlands, and the RIVM. The socio-economic status score per neighborhood, developed by Statistics Netherlands, provides a nuanced understanding of socio-economic disparities at the neighborhood level, making it a valuable tool for investigating the pandemic’s impact on different communities [24]. All COVID-19 vaccination data were obtained from the COVID Vaccination Information and Monitoring System (CIMS), provided by the RIVM, covering the period from January 6, 2021, to the end of 2022. Statistics Netherlands served as a reliable intermediary, facilitating the connection between datasets and upholding the privacy of the individuals involved, in compliance with the Dutch law (Statistics Netherlands Act 2003). No ethical board approval was necessary.

### Outcome, covariates and measurements

#### COVID-19-related hospital and ICU admission

COVID-19-related hospital and ICU admissions were provided by DHD. COVID-19-related hospital admissions were defined as admissions with ICD-10 codes U07.1 (confirmed COVID-19) or U07.2 (suspected COVID-19). Given the data structure, it was not possible to differentiate whether a patient had been hospitalized specifically due to COVID-19-related health issues or had been admitted for other health concerns while simultaneously having a COVID-19 infection. COVID-19-related hospital and ICU admissions were encoded binary, signifying the presence of at least one COVID-19-related hospital or ICU admission in the specific wave.

#### Covariates

Established on previous research, individual and neighborhood covariates were constructed [8–11, 14, 16, 17].

#### Individual characteristics

Individual characteristics were examined using standard items. Sex was measured as a dichotomous variable (1 = male; 2 = female). Age was measured on the first month of each wave, and classified into three categories (1 = 25–44 years; 2 = 45–64 years; and 3 = 65–79 years). Based on Statistic Netherlands’ classification, four categories for educational level were constructed: 1 = education missing (‘education unknown’); 2 = lower education (‘primary school’, ‘lower or preparatory vocational education’, and ‘lower general secondary education’); 3 = middle education (‘intermediate vocational education’, ‘higher general secondary education’ and ‘pre-university education, atheneum or gymnasium’); and 4 = higher education (‘higher professional education’ and ‘university’) [25]. Country of origin was categorized as the Netherlands, Europe (excluding the Netherlands), Suriname, Türkiye, Morocco and Other countries of origin, based on the most prevalent migration backgrounds in the region Haaglanden [26]. Household size was categorized as; 1 = single household; 2 = two-person household; 3 = three- or four-person household; and 4 = more than four-person household. Disposable household income was used, standardized for household size and composition. Financial wealth was defined as the difference between household assets and household debts. Both disposable household income and financial wealth were categorized into quintiles based on the study population, so all quintiles were the same size.

#### Neighborhood characteristics

For neighborhood-level characteristics, physical health, the socio-economic status score per neighborhood, and vaccination coverage were used.

##### Physical health

Physical health, defined as having one or more long-term conditions, was based on data obtained from the 2020 Health Monitors for Adults and the Elderly, conducted by 25 Municipal Public Health Services, Statistics Netherlands, and the RIVM. These data were modified at the neighborhood-level by the RIVM using the Small Area Estimates for Policy makers (SMAP) methodology [27], as the number of respondents was not large enough to provide robust neighborhood estimates or to be used at the individual level. The SMAP methodology relates participant responses to background characteristics such as age, sex, country of origin, household composition, educational level, income, and housing type. Additionally, the neighborhood of residence is taken into account. Using this model, the expected health of all adults is calculated, and these results are then aggregated to the neighborhood, district, and municipal levels. Physical health was divided into three categories based on a data-driven approach using the percentage of individuals per neighborhood reporting having one or more long-term conditions; good health = < 30%; moderate health = 30–35%; and poor health = > 35% [28].

##### Socio-economic status score per neighborhood (SES-WOA)

The neighborhood-level socio-economic status score was measured using the Socio-economic Status – Wealth, Education, and Employment History (SES-WOA) index developed by Statistics Netherlands [29, 30]. This composite indicator is based on three components: (1) financial welfare (income and wealth), (2) educational level (highest attained by the household’s reference person or partner), and (3) recent employment history of households over the previous four years. These components are combined into a total SES-WOA score at the household level. Statistics Netherlands then calculates a normalized average score per neighborhood. The score shows how a municipality, district or neighborhood scores compares to other municipalities, districts or neighborhoods on these three elements of SES. The greater the SES-WOA score is in a negative sense, the lower the SES is. The SES-WOA scores of the neighborhoods in the region Haaglanden are divided into three categories; high = > 0.2; middle = -0.2–0.2; and low = < -0.2, based on thresholds defined by Statistics Netherlands. This categorization was based on data-driven methods to ensure a roughly equal distribution of neighborhoods.

##### Vaccination coverage

The vaccination coverage at the neighborhood-level was based on CIMS data for the region Haaglanden from the RIVM. The vaccination coverage at the neighborhood-level encompasses all administered vaccination doses in the region Haaglanden up to July 12, 2021, shortly after the start of the third wave. This timing was chosen to account for the delay between vaccine administration and the development of immunity, and to assess the potential protective effect of vaccination during that wave. Vaccination coverage was included from the third wave onward, as vaccines became available to the general population at that time. In the second wave, the national vaccination campaign began with a phased rollout, initially targeting specific priority groups, such as older adults, individuals with underlying health conditions, and healthcare workers, limiting comparability at the population level. The vaccination coverage was categorized into two groups using data-driven methods: (1) vaccination degree = ≥ 60%; and (2) vaccination degree = < 60%. This cutoff resulted in an approximately equal distribution between the two categories and reflected meaningful differences in neighborhood-level coverage rates. The vaccination data, obtained from RIVM, were categorical and aggregated at the neighborhood level. The CIMS data includes vaccinations administered by Municipal Public Health Services, general practitioners, and other healthcare providers. It should be noted that additional vaccinations may have been administered by general practitioners or other healthcare providers without being recorded. Furthermore, data is only included in CIMS when explicit consent has been given for central registration. If an individual withdraws their consent, the previous registration in CIMS is eliminated.

### Statistical analyses

Statistical analyses were conducted in R, version 4.2.3. Descriptive data analyses were performed, examining differences in COVID-19-related hospital and ICU-admission rates among individuals aged 25–79 years in the region Haaglanden. Descriptive statistics for categorical variables were presented utilizing frequencies (n) and percentages (%), and mean values and standard deviation (SD) for continuous variables. To assess model performance and guide model selection, several diagnostic and goodness-of-fit measures were estimated for both univariate and multivariable analyses. These included the Akaike Information Criterion (AIC), Bayesian Information Criterion (BIC), deviances, and McFadden’s R2. Given that individuals were nested within neighborhoods and the study aimed to examine neighborhood-level disparities, the suitability of multilevel modelling was considered. Intraclass correlation coefficients (ICCs) were calculated to assess whether multilevel modelling was appropriate giving the structure and distribution of the data. All ICCs ranged from 0.106 to 2.33 × 10⁻¹⁶, indicating minimal variation at the neighborhood level. Therefore, logistic regression models with both individual and neighborhood characteristics were considered as the most appropriate approach. Univariate logistic regression analyses were performed to examine the association between each covariate and COVID-19-related hospital and ICU-admissions. Multivariable logistic regression analyses were performed to simultaneously assess the association between individual and neighborhood characteristics with COVID-19-related hospital and ICU admission. Multicollinearity was assessed by calculating the Variance Inflation Factors (VIF) in preliminary models that included individual independent variables. All VIF values ranged from 1.01 to 2.03, indicating a low level of multicollinearity among the included variables. All analyses were stratified for the three COVID-19 waves. As vaccination became available from January 2021 onwards, neighborhood vaccination coverage was only taken into account in the third wave. (Fixed effects) Coefficients were presented as odds ratios (OR) with 95% confidence intervals (CI). Variables with a significant p-value (p) < 0.05 were considered as potential risk factors for COVID-19-related hospital or ICU admission. Because of privacy regulations, cells with counts below 10 were masked in the results, and indicated as *n* < 10.

## Results

### Baseline characteristics of total population

A cumulative total of 721,234 individuals from the region Haaglanden were included in the first wave, 719,415 in the second wave, and 737,487 in the third wave. The sex distribution was nearly uniform across all waves. Most inhabitants were aged between 25-44 years, and 45–64 years old, with a mean age of approximately 49.6 years (SD = 14.9). The distribution of country of origin remained consistent across all waves. Households with more than four individuals were the least common. In terms of neighborhood characteristics, a middle SES-WOA score was most common (48.1%), and the majority of residents lived in a neighborhood with moderate health (30–35%). In the third wave, neighborhoods with vaccination coverage below 60% formed the largest group (66.2%). A comprehensive description of population characteristics per wave is provided in Table 1.

Over the entire study period, a total of 4,294 individuals were hospitalized due to COVID-19, of whom 881 required ICU care. Hospitalization rates varied across the three waves: 0.08% (*n* = 571) of the study population was hospitalized during the first wave, 0.40% (*n* = 2,865) during the second wave, and 0.17% (*n* = 858) during the third wave. For ICU, 0.02% (*n* = 159), 0.07% (*n* = 530) and 0.03% (*n* = 192) of the individuals were admitted respectively. The oldest individuals (aged 65–79 years) and males were most likely to be hospitalized and admitted to the ICU in all waves. Furthermore, individuals with lower educational levels, those in the lowest income and wealth quintiles, residents of neighborhoods with low SES-WOA scores, individuals of Moroccan origin, and those with poor physical health were proportionately more likely to be admitted to the hospital and ICU compared to their counterparts. The largest probability of admission by household size varied for hospital and ICU admission, and per wave. In the third wave, the majority of hospital and ICU admissions were from individuals living in neighborhoods with vaccination coverage below 60% (respectively 77.4% and 78.6%).

### Univariate analysis of COVID-19-related hospitalization

**Fig. 1:**
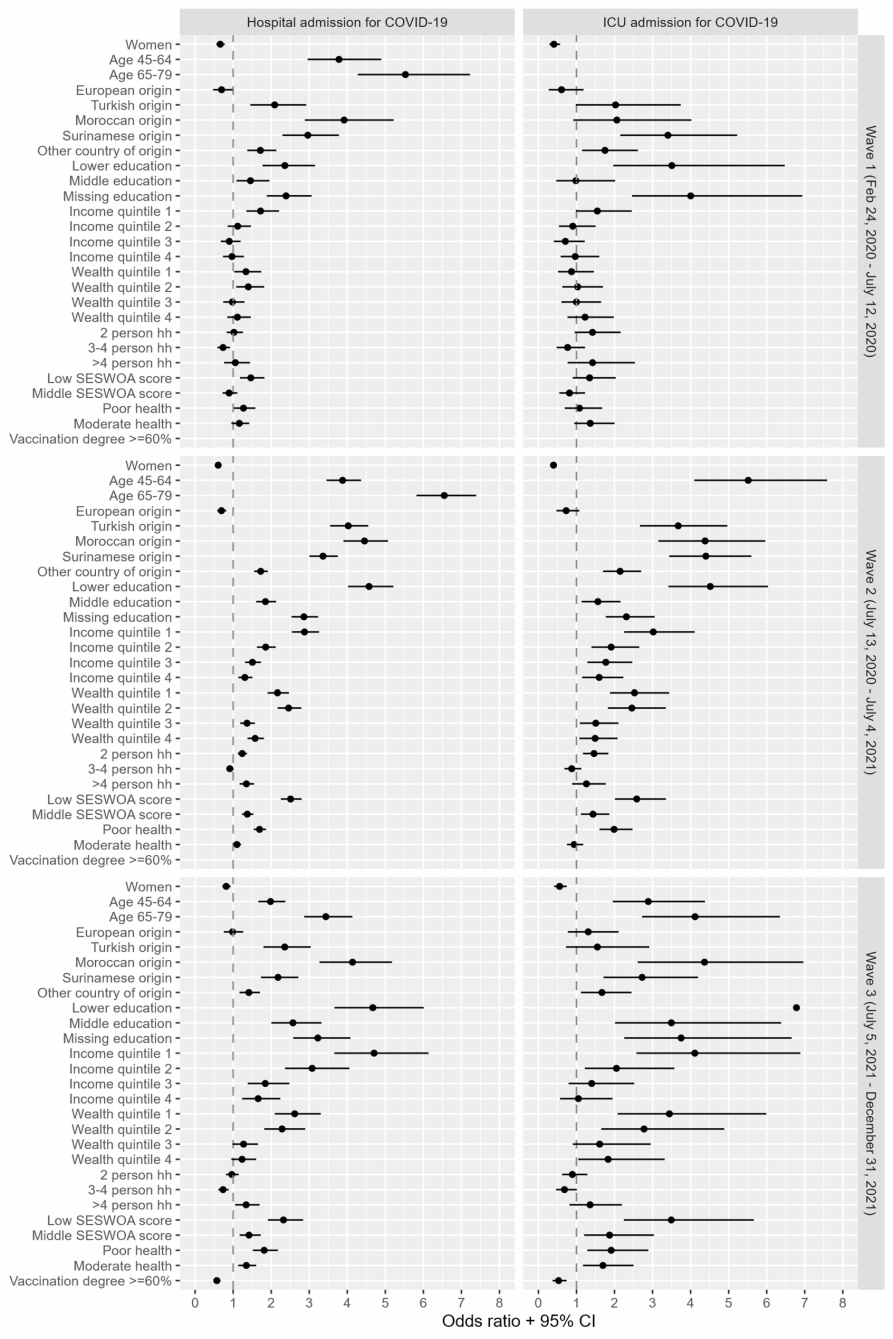
Univariate association between covariates and COVID-19-related hospital and ICU admissions per wave in region Haaglanden. *Notes*: CI: Confidence Intervals. hh: household size. The coefficients for age for ICU admissions fall outside the range. Wave 1: age 45–64 years (OR = 10.70, 95% CI: 5.52, 24.00, *p* <.05; and age 65–79 years (OR = 17.12, 95% CI: 8.73, 38.67, *p* <.05) Wave 2: age 65–79 years: (OR = 9.20, 95% CI: 6.80, 12.72, *p* <.05)

Figure 1 illustrates the ORs along with their 95% CIs from the univariate logistic regression models for each combination of covariates, hospital and ICU admissions, and wave. In all waves, being male, belonging to older age groups, having a non-European origin, not having a high educational level, having lower income or wealth, or residing in neighborhoods where individuals reported poor physical health with a higher deprived score, was associated with higher odds of COVID-19-related hospital admission. Only in the third wave, higher odds were observed for neighborhoods where individuals reported moderate physical health, or neighborhoods with vaccination coverage below 60%. The highest odds were observed for the oldest age group, non-Dutch country of origin, low educational level, and the lowest income quintile. The oldest age group had a 3.4 (95% CI: 2.9–4.1) to 6.5 (95% CI: 4.2–7.1) times higher odds of being hospitalized. Among all countries of origin, individuals of Moroccan origin showed the highest odds of hospitalization compared to Dutch origin, ranging from 3.9 (95% CI: 2.9–5.3) to 4.4 (95% CI: 3.9-5.0). The odds of hospitalization for individuals with a low educational level ranged from 2.4 (95% CI: 1.8–3.1) in the first wave to 4.7 (95% CI: 3.6-6.0) in the third wave. The odds for individuals in the lowest income quintile compared to the highest income quintile was higher in the third wave than the first and second wave (first wave: OR = 1.7 (95% CI: 1.3–2.2), second wave: OR = 2.9 (95% CI: 2.5–3.2), third wave: OR = 4.7 (95% CI: 3.6–6.1). See Additional file 1 for tables per wave with all regression coefficients.

### Univariate analysis of COVID-19-related ICU admission

In all waves, increased odds of COVID-19-related ICU admissions were observed among males, older age groups, most non-European country of origin, individuals with low and missing educational level, and in neighborhoods with vaccination coverage below 60% (see Fig. 1). Higher odds for individuals with lower income, lower wealth, and those living in neighborhoods with low or middle SES-WOA scores and with poor physical health were observed in the second and third wave. Older age was most strongly associated with higher odds of ICU admission, with ORs ranging from 2.9 (95% CI: 2.0-4.4) to 10.7 (95% CI: 5.5–24.0) for individuals aged 44 to 64 year and 4.1 (95% CI: 2.7–6.3) to 17.1 (95% CI: 8.7–38.6) for individuals aged 65–79 years, compared to individuals aged 25–44 years. Additionally, a low educational level in the second wave (OR = 4.5, 95% CI: 3.4-6.0) and third wave (OR = 6.8, 95% CI: 4.0-12.2), Surinamese origin in the second wave (OR = 4.4, 95% CI: 3.4–5.6), and Moroccan origin in the second wave (OR = 4.4, 95% CI: 3.2-6.0) and third wave (OR = 4.4, 95% CI: 2.6-7.0) were associated with the highest odds of ICU admission. See Additional file 2 for tables per wave with all regression coefficients.

**Table 1:** Descriptive information of population characteristics per wave in region Haaglanden, and stratified for COVID-19-related hospital and ICU admissions

Variable	Study Population (*N*, %)			Hospital Admissions (*N*, %)			ICU Admissions (*N*, %)		
	**Wave 1***n* = 721,234	**Wave 2***n* = 719,415	**Wave 3***n* = 737,487	**Wave 1***n* = 571 (0.08%)	**Wave 2***n* = 2.865 (0.40%)	**Wave 3***n* = 858 (0.17%)	**Wave 1***n* = 159 (0.02%)	**Wave 2***n* = 530 (0.07%)	**Wave 3***n* = 192 (0.03%)
**Mean age + SD** (years)	49.5 + 14.9	49.6 + 14.9	49.6 + 14.9	-	-	-	-	-	-
**Age group** (years)									
25–44	291,342 (40.4)	288,467 (40.1)	299,165 (40.6)	77 (13.5)	355 (12.4)	181 (21.1)	*n* < 10	48 (9.1)	32 (16.7)
45–64	289,411 (40.1)	289,026 (40.2)	292,171 (39.6)	289 (50.6)	1,374 (48.0)	361 (42.1)	*n* > 10	265 (50.0)	93 (48.4)
65–79	140,481 (19.5)	141,922 (19.7)	146,151 (19.8)	205 (35.9)	1,136 (39.7)	316 (36.8)	66 (41.5)	217 (40.9)	67 (34.9)
**Sex**									
Men	355,566 (49.3)	354,307 (49.2)	363,944 (49.3)	340 (59.5)	1,762 (61.5)	466 (54.3)	112 (70.4)	376 (70.9)	122 (63.5)
Women	365,668 (50.7)	365,108 (50.8)	373,543 (50.7)	231 (40.5)	1,103 (38.5)	392 (45.7)	47 (29.6)	154 (29.1)	70 (36.5)
**Country of Origin**									
Netherlands	432,416 (60.0)	431,335 (60.0)	435,062 (59.0)	255 (44.7)	1,173 (40.9)	395 (46.0)	73 (45.9)	200 (37.7)	83 (43.2)
Europe	77,895 (10.8)	76,840 (10.7)	81,943 (11.1)	32 (5.6)	145 (5.1)	68 (7.9)	*n* < 10	26 (4.9)	19 (9.9)
Türkiye	29,216 (4.1)	29,380 (4.1)	30,811 (4.2)	36 (6.3)	319 (11.1)	65 (7.6)	10 (6.3)	50 (9.4)	*n* < 10
Morocco	22,989 (3.2)	23,174 (3.2)	24,170 (3.3)	53 (9.3)	278 (9.7)	90 (10.5)	*n* < 10	47 (8.9)	20 (10.4)
Suriname	46,997 (6.5)	47,115 (6.5)	48,214 (6.5)	82 (14.4)	428 (14.9)	95 (11.1)	27 (17.0)	96 (18.1)	25 (13.0)
Other	111,721 (15.5)	111,571 (15.5)	117,287 (15.9)	113 (19.8)	522 (18.2)	145 (16.9)	33 (20.8)	111 (20.9)	*n* > 10
**Educational level**									
Missing	277,932 (38.5)	272,234 (37.8)	272,499 (36.9)	289 (50.6)	1,301 (45.4)	377 (43.9)	98 (61.6)	220 (41.5)	78 (40.6)
Low	100,348 (13.9)	100,783 (14.0)	102,603 (13.9)	103 (18.0)	768 (26.8)	209 (24.4)	31 (19.5)	159 (30.0)	44 (22.9)
Middle	150,032 (20.8)	151,929 (21.1)	159,440 (21.6)	95 (16.6)	470 (16.4)	182 (21.2)	13 (8.2)	83 (15.7)	54 (28.1)
High	192,922 (26.7)	194,469 (27.0)	202,945 (36.9)	84 (14.7)	326 (11.4)	90 (10.5)	17 (10.7)	68 (12.8)	16 (8.3)
**Disposable Household Income**									
Quintile 1	144,267 (20.0)	143,898 (20.0)	144,382 (19.6)	172 (30.1)	962 (33.6)	328 (38.2)	48 (30.2)	172 (32.5)	82 (42.7)
Quintile 2	144,267 (20.0)	143,903 (20.0)	144,620 (19.6)	112 (19.6)	622 (21.7)	215 (25.1)	28 (17.6)	109 (20.6)	41 (21.4)
Quintile 3	144,261 (20.0)	143,889 (20.0)	144,685 (19.6)	90 (15.8)	506 (17.7)	129 (15.0)	22 (13.8)	101 (19.1)	28 (14.6)
Quintile 4	144,231 (20.0)	143,874 (20.0)	144,732 (19.6)	97 (17.0)	439 (15.3)	116 (13.5)	30 (18.9)	91 (17.2)	21 (10.9)
Quintile 5	144,207 (20.0)	143,850 (20.0)	144,694 (19.6)	100 (17.5)	336 (11.7)	70 (8.2)	31 (19.5)	57 (10.8)	20 (10.4)
Missing	-	-	14,374 (2.0)	-	-	-	-	-	-
**Household Size**									
Single person household	170,150 (23.6)	168,767 (23.5)	174,347 (23.6)	146 (25.6)	622 (21.7)	218 (25.4)	34 (21.4)	109 (20.6)	51 (26.6)
Two person household	253,553 (35.2)	252,083 (35.0)	259,199 (35.2)	222 (38.9)	1148 (40.1)	314 (36.6)	72 (45.3)	238 (44.9)	68 (35.4)
3–4 person household	241,298 (33.5)	242,136 (33.7)	246,589 (33.4)	152 (26.6)	815 (28.4)	229 (26.7)	37 (23.3)	137 (25.8)	50 (26.0)
> 4 person household	56,233 (7.8)	56,429 (7.8)	57,352 (7.8)	51 (8.9)	280 (9.8)	97 (11.3)	16 (10.1)	46 (8.7)	23 (12.0)
**Financial Wealth**									
Quintile 1	144,293 (20.0)	143,908 (20,0)	147,518 (20.0)	131 (22.9)	724 (25.3)	267 (31.1)	27 (17.0)	149 (28.1)	62 (32.3)
Quintile 2	144,246 (20.0)	143,911 (20,0)	147,523 (20.0)	137 (24.0)	821 (28.7)	233 (27.2)	32 (20.1)	145 (27.4)	50 (26.0)
Quintile 3	144,258 (20.0)	143,892 (20,0)	147,510 (20.0)	96 (16.8)	457 (16.0)	130 (15.2)	31 (19.5)	89 (16.8)	29 (15.1)
Quintile 4	144,259 (20.0)	143,892 (20,0)	147,514 (20.0)	109 (19.1)	528 (18.4)	126 (14.7)	38 (23.9)	88 (16.6)	33 (17.2)
Quintile 5	144,178 (20.0)	143,812 (20,0)	147,422 (20.0)	98 (17.2)	335 (11.7)	102 (11.9)	31 (19.5)	59 (11.1)	18 (9.4)
**Physical Health**									
Poor health (> 35%)	188,280 (26.1)	187,433 (26.2)	190,638 (25.8)	166 (29.1)	1032 (36.0)	297 (34.6)	38 (23.9)	223 (42.1)	62 (32.3)
Moderate health (30–35%)	313,883 (43.5)	313,089 (43.5)	321,328 (43.6)	253 (44.3)	1120 (39.1)	369 (43.0)	80 (50.3)	176 (33.2)	92 (47.9)
Good health (< 30%)	219,071 (30.4)	218,893 (30.4)	225,521 (30.6)	152 (29.1)	713 (24.9)	192 (22.4)	41 (25.8)	131 (24.7)	38 (19.8)
**SES-WOA score**									
Low (<-0.2)	196,750 (27.3)	195,586 (27.2)	200,063 (27.1)	212 (37.1)	1228 (42.9)	343 (40.0)	58 (36.5)	227 (42.8)	86 (44.8)
Middle (-0.2-0.2)	346,566 (48.1)	345,812 (48.1)	354,431 (48.1)	228 (39.9)	1190 (41.5)	377 (43.9)	62 (39.0)	223 (42.1)	83 (43.2)
High (> 0.2)	177,918 (24.7)	178,017 (24.7)	182,993 (24.8)	131 (22.9)	447 (15.6)	138 (16.1)	39 (24.5)	80 (15.1)	23 (12.0)
**Vaccination Coverage**									
Vaccination degree < 60%	-	-	488,084 (66.2)	-	-	664 (77.4)	-	-	151 (78.6)
Vaccination degree ≥ 60%	-	-	249,403 (33.8)	-	-	194 (22.6)	-	-	41 (21.4)

Notes: SD: Standard Deviation Cells with counts below 10 were masked and indicated as *n* < 10. To prevent potential disclosure, additional cells in the cross-tabulations with counts above 10 were also masked

### Multivariable analysis of COVID-19-related hospital admission

**Fig. 2:**
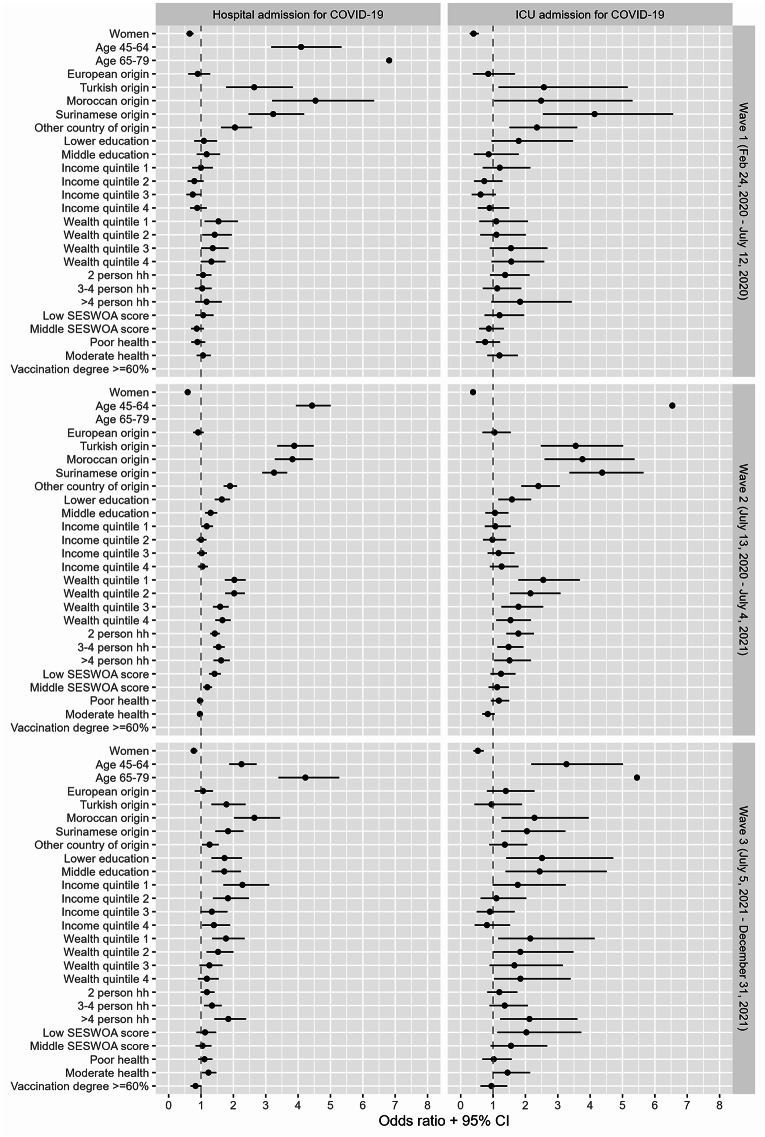
Multivariable associations for COVID-19-related hospital and ICU admission per wave in region Haaglanden. Notes: CI: Confidence Intervals. hh: household size. For COVID-19-related hospital and ICU admissions, the coefficients for age fall outside the range. Hospitalization wave 2: age 65–79 years (OR = 9.44, 95% CI: 8.25, 10.83, *p* <.05). ICU admission wave 1: age 45–64 years (OR = 9.41, 95% CI: 4.79, 21.32, *p* <.05); and age 65–79 years (OR = 15.42, 95% CI: 7.42, 36.33, *p* <.05). ICU admission wave 2: age 65–79 years: (OR = 13.87, 95% CI: 9.91, 19.74, *p* <.05). Variance Inflation Factors (VIF) values ranged from 1.01 to 2.03 in preliminary models using individual variables

Figure 2 shows the ORs along with their 95% CIs from the multivariable logistic regression models for each admission type and wave. After adjusting for all other covariates, the odds of being hospitalized remained statistically significantly higher for older age, males, and non-European country of origin in all waves. In the first wave, educational level, income quintile, household size, SES-WOA, and physical health score of the neighborhood were not statistically significantly associated with hospital admission. In the second wave, individuals living in neighborhoods with low and middle SES-WOA scores were more likely to be admitted to the hospital. During the first and second waves, individuals in the lower wealth quintiles had significantly higher odds of COVID-19-related hospitalization, while in the third wave, this association remained significant for those in the first and second quintiles specifically. In both the second and third waves, low and middle educational levels, the lowest income quintile, and household size were associated with higher odds of hospitalization. Furthermore, vaccination degree did not statistically significantly correlate with hospitalization rates. See Additional file 3 for tables per wave with all regression coefficients.

### Multivariable analysis of COVID-19-related ICU admission

Similar to the multivariable analysis of hospital admissions, the odds of being admitted to the ICU were statistically significantly higher for older age, males, and most non-European country of origin in all waves (see Fig. 2). In the first wave, none of the other covariates were statistically significantly associated with ICU admission. In the second wave, individuals in lower wealth quintiles had higher odds of ICU admission. In the second and third wave, low educational level and multiple person households (except for 2-person and 3–4 person households in the third wave) were associated with higher odds of ICU admission. In the third wave, individuals living in neighborhoods with low SES-WOA scores and those from neighborhoods with moderate physical health had higher odds of being admitted to the ICU. See Additional file 4 for tables per wave with all regression coefficients.

## Discussion

This research sought to examine the association between demographic, socio-economic, and health characteristics, including vaccination coverage, and COVID-19-related hospital and ICU admissions, with a particular focus on neighborhood differences in the region Haaglanden, the Netherlands. Over the entire study period, 4,294 individuals were hospitalized due to COVID-19, of whom 881 required ICU care. Higher odds of hospital and ICU admissions were found among older individuals, men, those with lower educational levels, individuals of Moroccan origin, those with lower income and wealth, poor physical health, and residents of low SES-WOA neighborhoods. In the third wave, the majority of the population (66.2%) lived in neighborhoods with vaccination coverage below 60%, which also accounted for the highest proportions of hospital (77.4%) and ICU admissions (78.6%), highlighting an overrepresentation of residents with low vaccination coverage among hospital and ICU admissions, and underscoring the significance of vaccination coverage in mitigating severe outcomes. The proportion of individuals admitted to the hospital and ICU due to COVID-19 was highest during the second wave, likely due to both the greater number of infections and the prolonged duration of this wave compared to the first and third waves. This can be explained by the fact that higher infection rates within specific population groups directly increase the risk of hospital and ICU admissions, as a greater number of infections leads to an increased amount of severe cases requiring medical care.

The multivariable analysis indicated that older age groups and non-Dutch country of origin, excluding European origin, are associated with an increased risk of COVID-19-related hospital or ICU admission. This finding is consistent with previous research, as well as the observed association with gender [8–11, 17, 31, 32]. Even after adjusting for physical health, income, and financial wealth, the differences in admission rates by country of origin remained significant, suggesting the presence of additional contributing factors. These may include specific underlying comorbidities, and lifestyle factors such as smoking and obesity, which are more prevalent in these groups [14, 16]. In addition to the factors examined in this study, other determinants may influence the risk of hospitalization. Research in the Netherlands showed that individuals with limited health literacy or those facing cultural and language barriers may be more likely to not (fully) understand (health) information and/or delay seeking medical care, potentially resulting in poorer health outcomes and higher hospitalization rates [33–36]. Moreover, genetic predispositions and chronic stressors, including psychosocial and environmental influences, may also contribute [33–36]. Although these factors were not captured in our registry-based study, they are likely significant contributors and should be further explored in future research.

Furthermore, the results of this study indicated that neighborhood characteristics were associated with higher odds of hospital and ICU admissions. In line with previous research [10], higher odds of COVID-19-related hospital admissions were observed in neighborhoods with low SES-WOA scores during the second wave, and this trend extended to higher odds of ICU admissions during the third wave. Over the total study period, between 37.1% and 42.9% of hospital admissions and 36.5 to 44.8% of ICU admissions originated from neighborhoods with low SES-WOA scores, while 27.1 to 27.3% of the study population lived in these neighborhoods (see Table 1). After adjusting for the other characteristics, part of the SES-WOA association observed in the univariate analysis was explained by these factors. However, a neighborhood-related association with SES-WOA score remained, suggesting that these neighborhood characteristics may still independently contribute to the observed outcomes. Initially, the spread of COVID-19 in the Netherlands followed a different pattern, with little to no significant difference between the neighborhoods. At the onset of the pandemic (the start of the first wave), infections in the Netherlands were partly introduced by individuals returning from ski vacations or other international travels, activities primarily affordable for those from higher-income neighbourhoods [37]. As the pandemic evolved, this dynamic shifted: from July 2020 (the start of the second wave) onwards, despite preventive measures, the burden of COVID-19 hospital admissions became more prevalent in neighborhoods with low SES-WOA scores.

Moreover, a higher risk of COVID-19-related hospitalization and ICU admissions was also observed among individuals with lower individual socio-economic indicators, such as lower education, lower disposable household income, and lower wealth, aligning with previous findings [11, 17]. In addition to these individual socio-economic inequalities, household composition plays a significant role in increasing vulnerability to COVID-19 [11]. A higher likelihood of hospitalization may be linked to a greater probability of infection. Individuals in lower SES groups often face heightened exposure risks due to structural disadvantages [11, 17, 32]. For example, individuals with lower education levels and lower incomes are more likely to hold lower-status jobs that cannot be performed remotely, which increases their contact with others and makes it more difficult to adhere to COVID-19 guidelines [38–40]. Moreover, smaller and more crowded living spaces in these communities may facilitate easier transmission of the virus within households [41]. Previous research has shown that multi-person households are at risk for severe outcomes, as maintaining physical distancing and preventing intra-household transmission are more challenging in such settings [41–43]. Our findings further confirm that individual socio-economic indicators, along with household composition, significantly contribute to the observed disparities in hospital and ICU admissions.

In addition to the risk of infection, the severity of the disease course after infection also determines hospital and ICU admission rates. Previous research indicated that individuals with underlying health conditions, such as cardiovascular diseases, diabetes, and respiratory conditions, may be at higher risk of experiencing severe COVID-19 symptoms [44, 45]. Furthermore, Kunst and colleagues (2021) found that poorer self-reported health increases the likelihood of hospitalization [17]. In contrast, in the current study, reporting physical health at the neighborhood level was only significantly associated with hospital admissions during the third wave, with higher odds observed in neighborhoods where individuals reported physical health as moderate. However, no association was found between physical health and ICU admissions, which may be due to limited variation in health across neighborhoods. Additionally, neighborhood-level average health does not accurately reflect individual health.

During the second and third wave, vaccinations became widely available to the general population, aiming to reduce both infection transmission and symptoms after infection. Vaccinations can protect the vaccinated individual and, with sufficiently high vaccination rates, may contribute to herd immunity at the neighborhood level [46]. However, in the current study, no association was found between vaccination coverage and hospital or ICU admission. This contradicted the intended protective effects of vaccination and previous research [47, 48]. Possible explanations for this discrepancy include delays in achieving sufficiently high vaccination rates for herd immunity, variations in vaccination uptake across demographic groups, and differences in vaccine effectiveness against emerging variants. Another possible explanation is that in our study, no neighborhood reached the vaccination threshold considered necessary for herd immunity, such as the 90% target recommended by the World Health Organization [49]. Therefore, we used a threshold of 60% to categorize vaccination coverage. Observations from hospitals indicated that primarily unvaccinated individuals were being admitted. This may reflect gaps in vaccination uptake, particularly among vulnerable groups [50, 51].

### Strengths and limitations

This research study has several notable strengths. To the best of our knowledge, this is the first study investigating the association between various characteristics, including vaccination coverage, and COVID-19-related hospital and ICU admissions, with a specific focus on neighborhood differences in the region Haaglanden, the Netherlands. Additionally, the inclusion of the entire regional population minimizes selection bias, enhances the robustness and generalizability of the findings across all pandemic waves. Furthermore, the recalculation of the population size at the start of each wave ensures the accuracy and relevance of the study’s findings across different time periods.

Despite the strengths of this study, several limitations should be considered. First of all, the cross-sectional design of the study limits the ability to establish causal relationships and does not allow for tracking changes in individual health status, hospital and ICU admissions, or other socio-economic circumstances over time. As a result, interim developments or repeated events during the study period may have not been fully captured. Secondly, the reliance on registration data limited the ability to confirm whether COVID-19 was the primary reason for hospital and ICU admissions, particularly in cases involving patients with multiple comorbidities. Nevertheless, during the first wave, hospital admissions were likely dominated by COVID-19 cases, as routine hospital care for other diseases was largely suspended [52]. Additionally, the reliance on registry data prevented testing all possible mechanisms for higher hospitalization and ICU rates. Thirdly, neighborhood-level SMAP data on physical health were used as a proxy for underlying morbidity. Individual-level data on specific health conditions, such as diabetes or chronic lung diseases [45], known contributors to severe COVID-19 outcomes, would have provided greater precision and insight compared to the broader neighborhood-level measure used in this study. It is also possible that the used SMAP data partially reflect the impact of COVID-19 and its linked public health measures because it is self-reported data collected during the COVID-19 pandemic [27]. These data may differ from municipal or national statistics due to variations in computation methodologies, incorporating more background and neighborhood characteristics. Therefore, careful interpretation of these figures is essential. Furthermore, vaccination data were available only at the neighborhood-level, limiting the ability to analyze individual vaccination status and its direct impact on hospitalization or ICU admission risk. Lastly, individuals aged 80 years and older were excluded from the analyses, but accounted for many severe COVID-19 cases. Further research should examine the determinants of hospital and ICU admission for this group, and how these might differ for nursing home residents and elderly with pre-pandemic poorer health.

## Conclusion, implications for further research and practice

To conclude, this study highlights that both individual and neighborhood-level factors were associated with an increased risk of COVID-19-related hospital and ICU admissions, with the individual risk factors often concentrated in neighborhoods with low socio-economic status scores. Future research should explore how local environmental factors, community health resources and healthcare accessibility collectively shape health disparities during public health crises.

In terms of public health strategies, pandemic preparedness plans should prioritize high-risk individuals and adjust the guidelines with and for these high-risk individuals to ensure suitability, rather than focusing exclusively on disadvantaged neighborhoods. However, since high-risk individuals are overrepresented in socio-economically disadvantaged areas, identifying these areas early on remains essential for effective resource allocation during outbreaks. Socio-economic vulnerabilities, rather than case incidence alone, should guide the allocation of resources, as these areas often house populations more vulnerable to severe outcomes. The findings of this study highlight the need for targeted interventions, such as culturally tailored vaccination campaigns and efforts to improve healthcare access and reduce vaccine hesitancy. Collaborating with trusted local figures and leveraging community networks can increase effectiveness of these efforts. Finally, studying the role of community health resources and regional policies could provide valuable insights into reducing disparities in future public health emergencies.

## Electronic supplementary material

Below is the link to the electronic supplementary material.

Supplementary Material 1

Supplementary Material 2

Supplementary Material 3

Supplementary Material 4

## Data Availability

This study was conducted using microdata sets of Statistics Netherlands (CBS) (https://www.cbs.nl/en-gb/our-services/customised-services-microdata/microdata-conducting-your-own-research). The data that support the findings of this study are available from Statistics Netherlands but restrictions apply to the availability of these data, which were used under license for the current study, and so are not publicly available. Data are however available from the corresponding author upon reasonable request and with permission of Statistics Netherlands.

## Abbreviations

AIC: Akaike Information Criterion
BIC: Bayesian Information Criterion
CIMS: COVID Vaccination Information and Monitoring System
COVID: 19-Coronavirus disease 19
DHD: Dutch Hospital Data
ICU: Intensive Care Unit
RIVM: National Institute for Public Health and the Environment
SARS-CoV-2: Severe acute respiratory syndrome-coronavirus
SES: Socio-economic status
SES-WOA: Status score per neighborhood based on financial wealth, education level and employment history
SMAP: RIVM Small Area Estimates for Policy makers
VIF: Variance Inflation Factor
